# Spotted Fever Group Rickettsioses in Central America: The Research and Public Health Disparity among Socioeconomic Lines

**DOI:** 10.3390/insects13080674

**Published:** 2022-07-27

**Authors:** Kyndall C. Dye-Braumuller, Marvin S. Rodríguez Aquino, Stella C. W. Self, Mufaro Kanyangarara, Melissa S. Nolan

**Affiliations:** 1 Department of Epidemiology and Biostatistics, Arnold School of Public Health, University of South Carolina, Columbia, SC 29208, USA; kyndallb@email.sc.edu (K.C.D.-B.); scwatson@mailbox.sc.edu (S.C.W.S.); mufaro@mailbox.sc.edu (M.K.); 2 Centro de Investigación y Desarrollo en Salud, Universidad de El Salvador, San Salvador CP1101, El Salvador; marvin.rodriguez@ues.edu.sv

**Keywords:** *Rickettsia* spotted fever group *Rickettsia*, tick-borne disease, Central America, El Salvador, Honduras, Nicaragua, Guatemala, neglected diseases, poverty

## Abstract

**Simple Summary:**

Tick-borne diseases are an important public health issue globally. Spotted fever group rickettsioses (SFGR), a group of related tick-borne pathogens, can cause significant disease, but is widely underreported and likely misdiagnosed. In Central America, there is little known about SFGR, and there seems to be a socioeconomic-related difference between the most-developed and least-developed countries in the region. The least-developed countries (El Salvador, Guatemala, Honduras, and Nicaragua) disproportionately underreport research or studies regarding SFGR disease compared to the most-developed countries (Belize, Panama, and Costa Rica). We compared human development and poverty indicators among Central American countries for this review of SFGR *Rickettsia*-related articles. The least-developed countries are at a higher risk or are more vulnerable to SFGR disease than the most-developed countries in this region.

**Abstract:**

Tick-borne diseases including rickettsial diseases are increasing in incidence worldwide. Many rickettsial pathogens can cause disease which is commonly underdiagnosed and underreported; *Rickettsia* pathogens in the spotted fever group (SFGR) are thus classified as neglected bacterial pathogens. The Central American region shoulders a large proportion of the global neglected disease burden; however, little is known regarding SFGR disease here. Although development varies, four of the seven countries in this region have both the highest poverty rates and SFGR disease burdens (El Salvador, Honduras, Guatemala, and Nicaragua), compared to Belize, Panama, and Costa Rica. Utilizing the Human Development Index (HDI), we compared published articles related to SFGR *Rickettsia* prevalence in the lowest-HDI-scoring countries to the highest-HDI-scoring countries. Our analysis identified a distinct dichotomy in publication, and by proxy, potentially awareness and knowledge of SFGR tick-borne disease in Central America, where the least-developed countries are at the highest risk for, yet the most vulnerable to, SFGR disease.

## 1. Introduction to Spotted Fever Group Rickettsioses

Spotted fever group rickettsioses (SFGR) are a group of diseases caused by bacteria in the genus *Rickettsia.* Rickettsial diseases are emerging and increasing in incidence worldwide [1], and the SFGR group is responsible for the majority of known pathogenic *Rickettsia* species [2,3]. All SFGR pathogens are transmitted to vertebrate animal hosts, including humans, from the bite of an infected hard tick (Acari: Ixodidae). SFGR bacteria have a worldwide distribution: human disease has been documented on almost every continent, with a significant focus in the subtropical and tropical regions of the world [4,5,6,7]. Clinical disease can vary depending on the species of *Rickettsia*, along with access to treatment and co-morbidities; however, once treatment is administered, disease is typically halted, and recovery is swift [8]. Notable species that can cause significant morbidity and high mortality in humans include *Rickettsia rickettsii*, the causative agent for Rocky Mountain spotted fever (RMSF); *R. africae*, the causative agent for African tick-bite fever; and *R. conorii*, the causative agent for both Mediterranean spotted fever and Israeli spotted fever. Because symptoms, signs, and severity can vary between rickettsial species, it is difficult to distinguish SFGR infections from other common infections—especially those that can cause a fever-like illness with undifferentiated symptoms [8]. These tick-borne SFGR are thus commonly misdiagnosed and underreported, and as such, have been classified as neglected bacterial pathogens [9,10,11].

Central America has a history of various infectious diseases impacting large numbers of people, notably many neglected tropical diseases (NTDs) such as Chagas disease, leishmaniasis, and dengue [12,13,14]. Despite the wide range of potential hosts and suitable tropical habitat for ticks in this region, information on tick-borne disease risk, including SFGR, risk factors, ecology, and epidemiology, is limited [15,16]. Fifteen tick-borne pathogens have been documented in the Central America and Caribbean region, approximately half of which are in the SFGR group [15]. Approximately 80 species of ticks have been documented in the region, many of which are competent pathogen vectors [16]. However, given the vast biodiversity of fauna (both ticks and potential hosts) and poor public health systems in this region, it is thought that the number of pathogens circulating in this environment and human populations is a severe underestimation [15].

The link between poverty and NTDs has been described globally, including in the Central American region: where populations with the most extreme poverty are disproportionately impacted by NTDs [12,13]. Despite the knowledge of certain established NTDs in the region, the impact and geographic distribution of tick-borne diseases, including SFGR, is largely underdescribed in the scientific literature. We hypothesized that the least-developed Central American countries have placed ticks and tick-borne disease research, awareness, and surveillance on the proverbial backburner as their resource-limited public health departments combat other NTDs. This review aimed to compare various indicators of development, including the United Nations Human Development Index (HDI), across the Central American region, along with the history of tick-borne SFGR disease research, surveillance, or knowledge to understand the vulnerability of populations in this region to these diseases.

## 2. The United Nations Human Development Index

The HDI is a composite measure developed by the United Nations Development Programme to assess multiple factors regarding human development: health, education, and standard of living. The health dimension is assessed by life expectancy at birth; the education dimension is assessed by mean expected years of schooling for children and mean years of schooling for adults over 25 years old; and the standard of living dimension is measured by gross national income per capita [17]. The HDI has been shown as a reliable indicator of human development for ecological comparisons between countries—especially in relation to infectious diseases. Lower HDI scores (indicating less developed countries) have been significantly linked with increased malaria incidence globally [18], higher dengue mortality in Latin America and the Caribbean [19], and higher leishmaniasis mortality in Brazil [20].

As shown in Table 1, most Central American countries are scored as “Medium” HDI countries (El Salvador, Guatemala, Nicaragua, and Honduras), Belize is considered a “High” HDI country, and both Panama and Costa Rica are scored as “Very high” HDI countries. In general, those countries ranked as “Medium” have higher percentages of child malnutrition, higher percentages of total populations vulnerable to multidimensional poverty and below the national poverty line, and higher percentages living at less than USD 2/day. Overall, Guatemala and Honduras have the highest proportion of those in poverty and child malnutrition. Not coincidentally, these four countries with the worst (lowest) HDI scores contribute the most cases of NTDs reported in the region annually in comparison to those countries with better (higher) HDI scores [13]. We anticipate that in agreement with this pattern, more knowledge, surveillance, or research related to tick-borne diseases will be evident in those countries ranked with higher HDI scores compared to the bottom four (El Salvador, Guatemala, Nicaragua, and Honduras).

## 3. Systematic Review: SFGR in Central America

### 3.1. Search Terms

A comprehensive systematic literature review was conducted using the following databases: PubMed, Web of Science, SciELO, and Latin American and Caribbean Health Sciences Literature. Additional articles were recovered from references of selected articles when applicable and searches within bachelor’s and master’s thesis databases from public universities in the selected countries. There were no limits set for publication date, although more recent articles (e.g., published within the past 10 years) were favored. The following search terms were used to locate studies evaluating the presence of SFGR bacteria in humans, animals, and ticks, as well as the serology results of humans or animals for antibodies to SFGR in the selected countries: [country name] AND Rickettsia OR tick surveillance.

A total of 274 publications were initially accumulated from all databases. Following title and abstract review, 46 duplicates and 84 non-relevant publications were removed for multiple reasons including: no mention of SFGR bacteria or SFGR disease. The remaining 73 publications were reviewed, and 4 additional publications were added from references. Following the complete read-through, six publications were removed as four were review articles with no original content, one used the same sample population as another article, and one did not test for SFGR pathogens. The remaining 71 publications were used for the following literature review.

### 3.2. Search Results from High- vs. Low-HDI Countries

As shown in Table 2, there is a sharp distinction between Panama and Costa Rica and the remaining countries regarding SFGR *Rickettsia*, with 24 and 27 articles published, respectively. Only three articles have been published regarding SFGR *Rickettsia* from Belize. In contrast, those countries with a lower HDI score and higher rates of poverty (El Salvador, Guatemala, Honduras, and Nicaragua) have six or fewer articles published each. Further, those resource-limited countries’ published manuscripts addressing SFGR *Rickettsia* have a much lower percentage of authors from the original country compared to Panama and Costa Rica (Supplementary Table S1). The majority of El Salvador, Belize, Nicaragua, Honduras, and Guatemala manuscripts’ authors are from foreign counties. This strengthens the suggestion that Panama and Costa Rica researchers, physicians, etc., can possibly secure funding from their own government, garner local support, or use their own technological resources for this research and tick-borne disease awareness. The subsequent paragraphs will review the scientific literature of these four impoverished, resource-limited countries to expose potential themes of SFGR transmission among their vulnerable populations.

**Figure 1:**
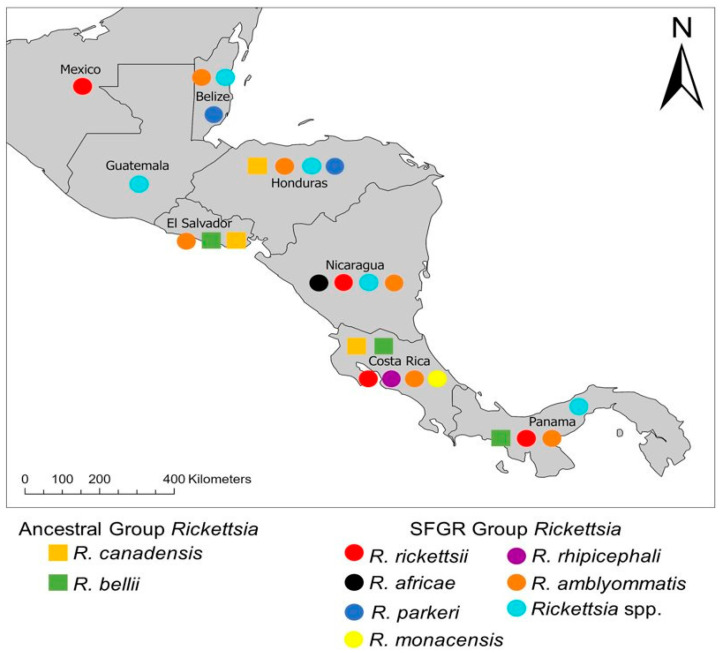
Current known geographical distribution of *Rickettsia* spp. in Central America.

Figure 1 depicts the geographical biodiversity of SFGR and ancestral group *Rickettsia* species in Central America from these articles and additional reviews [4,5,15,24]. To keep things simplistic, we did not include *Rickettsia* species from the typhus group (*R. prowazekii* and *R. typhi*).

### 3.3. El Salvador

The first SFGR investigation in El Salvador was published in 1993 when the World Health Organization (WHO) conducted a global serological investigation into human rickettsial diseases in the early 1990s. Forty human serum samples from patients with undifferentiated fever were submitted to this study from El Salvador, and thirteen (32.5%) were positive through an indirect fluorescence antibody (IFA) assay for SFGR antibodies [25]. Although identification of the SFGR species was not conducted, the WHO concluded that this study demonstrated the extensive spread of rickettsial infection and the need to continue surveillance [25]. A second global serological investigation was conducted around the same time looking at rickettsial antibodies in humans and animals using both an enzyme-linked immunosorbent assay (ELISA) and IFA. Sixteen (40.0%) of the forty human samples were positive by ELISA to SFGR antibodies and thirteen (32.5%) were positive by IFA to *R. conorii* antibodies from El Salvador [26]. The positive samples for *R. conorii*, the causative agent for both Mediterranean and Israeli spotted fevers (not known in the Central American region), was noted as most likely an indication of cross-reactivity for SFGR antibodies. The authors concluded that the relatively high prevalence of antibodies in El Salvador compared with the relatively few reports of clinical disease lead to the probability that cases were not recognized or not reported in this country [26].

The next investigations into SFGR in this country were not reported until nearly 20 years later: when a singular investigation into ticks parasitizing turtles was published in 2012. This investigation was the first published report to document a species of tick and rickettsial bacteria in the country. Four *Amblyomma sabanerae* ticks found on a turtle in the western portion of the country were all positive for *R. bellii* and negative for any SFGR bacteria [27]. Although *R. bellii* is not in the SFGR group, this is a significant report as the first documentation of *Rickettsia* in the country. No environmental risk factors for infection in humans were noted.

In both 2014 and 2021, two wide-scale tick and tick-borne disease surveillance investigations were published increasing the number of known Salvadoran tick species from 1 to 12 and increasing the number of documented *Rickettsia* species from 1 to 3. Both studies collected ticks from peridomestic and livestock animals for identification and analysis, then tested select ticks for the presence of *Rickettsia* through PCR followed by sequencing. The 2014 study did not report exact percentages of ticks positive for *Rickettsia*; however, of the 250 ticks tested, 3 tick species were positive for the SFGR group species *R. amblyommatis* (*A. auricularium*, *A. cajennense*, and *A. parvum*), two positive for the ancestral group species *R. canadensis colombianensi* (*A. dissimile* and *A. scutatum*), and two positive for *R. bellii* (*A. dissimile* and *A. ovale*) [28]. The 2021 study expanded tick collections across the entire country, collecting 1181 ticks, and tested a large majority of them for pathogens [29]. *Rickettsia amblyommatis* was found in 10/13 (77%) *A. mixtum* ticks, 8/16 (50%) *A. parvum* ticks, 1/9 (11%) unknown *Amblyomma* spp. nymphal ticks, and in 1/13 (8%) *Dermacentor nitens* ticks. *R. canadensis colombianensi* was found in 3/31 (10%) *A. dissimile* ticks and in 2/18 (11%) *A. scutatum* ticks. *R. bellii* was found in 1/31 (3.2%) *A. dissimile* ticks and in 1/6 (17%) *A. ovale* ticks. Of note for the 2021 study was that multiple ticks were collected from humans—both *Rhipicephalus sanguineus* and *R. microplus*—but these were negative for any SFGR pathogens.

### 3.4. Guatemala

Only one publication reports the presence of SFGR bacteria in Guatemala, published in 2013. This study documents an outbreak investigation conducted in 2007 during which 17 persons from a farming community in the southeastern Department of Jutiapa reported an acute febrile illness, of which 2 people died [30]. Sixteen of the seventeen patients were tested through IFA, Western Blot, and PCR to detect acute rickettsial infection. Seven of the sixteen tested (40%) were positive for both IgG and IgM SFGR antibodies, and one individual (6.25%) was positive by PCR for SFGR DNA. The study concluded that 10/16 tested individuals were confirmed or probable cases of SFGR. In addition, 97 ticks were collected from the area and tested for SFGR DNA through PCR. Of the 12, 1 (8.3%) *A. cajennense* tick and 0/85 *R. microplus* ticks were positive for SFGR DNA. Although the authors did not determine the species of SFGR bacteria, they do comment that the most likely culprit was *R. rickettsii* or *R. africae* [30]. Additionally, risk factors noted for all patients included contact with animals such as dogs and rats, and a majority of the cases were farm workers and had seen ticks on animals routinely.

### 3.5. Honduras

A 1971 study documents the first known presence of SFGR in Honduras after a large Central American human serological survey. Samples from El Salvador, Honduras, Guatemala, and Nicaragua were submitted; however, only samples from Honduras and Nicaragua were positive for SFGR antibodies [31]. Samples were tested through complement-fixation (CF) and microscopic agglutination tests (MA); of the 348 samples submitted from Honduras, 3 (0.9%) and 1 (0.3%) were positive for SFGR antibodies through CF and MA, respectively. The location of patients who submitted samples was not given, and no risk factors were identified from the data.

The next documentation related to SFGR in Honduras was reported 40 years later, when a single tick-bite investigation was initiated regarding a US traveler who developed SFGR disease after reporting a tick bite from Honduras during his stay [32]. The man reported typical SFGR symptoms including erythema, flu-like symptoms, fever, headache, and a possible eschar at the tick bite site. He was positive by serology, and the authors suggested *R. rickettsii* as the causative agent. Risk factors included reported frequent contact with horses and dogs, as well as spending a lot of time outdoors in Honduras.

Two additional SFGR serological studies were published in 2010 and 2021 regarding risk to US military personnel stationed in Honduras (locations not described). The first study investigated the presence of antibodies to *R. rickettsii* in feral cats that frequently came into contact with military personnel on base [33]. Only 12 cats were tested through IFA, and 2 (16%) were positive for *R. rickettsii* antibodies. Because these cats were being handled by troops, the authors noted that close contact with feral animals increased the risk for exposure to this tick-borne SFGR, although no ticks were found [33]. The second study investigated 1000 US military personnel deployed in Honduras for greater than 6 months between 2000 and 2016. Included participants were evaluated pre- and post-deployment to Honduras for antibodies to SFGR through both MA and IFA [34]. Results revealed 39/1000 (3.9%) personnel seroconverted while on deployment to Honduras, and 9 of these 39 individuals were also positive for the antibodies of 1 or 2 additional pathogens [34]. Minority race was associated with increased rate of SFGR seroconversion, and all military personnel spent copious amounts of time outdoors.

Lastly, a tick surveillance investigation was conducted in the northwestern portion of Honduras in 2014 where ticks were collected from animals and humans and tested for SFGR bacteria presence through PCR [35]. SFGR group *R. amblyommatis* was found in 4/9 (44.4%) *A. mixtum* ticks (found biting humans), 1/9 (11.1%) *A. longirostre* ticks, and 12/67 *Amblyomma* spp. larval ticks. Ancestral group *R. canadensis colombianensi* was found in 3/12 (25%) *A. dissimile* and *Amblyomma* spp. larval ticks. The authors made note that many of the people handling animals found ticks on themselves, and animal contact was a notable risk factor for exposure [35]. This study was the first to document *R. amblyommatis* and *R. canadensis colombianensi* in Honduras.

### 3.6. Nicaragua

The same 1971 Central American serological study mentioned for Honduras reported that only 1/312 (0.3%) patients tested positive for SFGR antibodies through CF only in Nicaragua [31]. No other reports were published from Nicaragua regarding SFGR bacteria or disease until the late 2010s.

One such study, a human serological investigation, was conducted from 2008 to 2009 through enrolling febrile patients with undiagnosed febrile illness from hospitals. Patient sera was tested for IgG antibodies to *R. rickettsii* through ELISA, followed by IFA for confirmation and PCR to confirm acute infection: results indicated that 51/748 (6.8%) of patients had antibodies to *R. rickettsii* from ELISA only and 6 acute infected patients with *R. rickettsii* were identified through PCR [36]. Risk factors associated with SFGR antibodies or SFGR infection included rural residence, less education, river water exposure, exposure to farm animals and domestic pets, and exposure to ectoparasites [36].

Three additional studies carried out between 2013 and 2016 investigated the health of dogs in relation to humans and the risk of rickettsial infection in Nicaragua. The first, conducted near the north-central border of Nicaragua and Honduras, focused on only ticks from peridomestic and domestic dogs. Of these community dogs, 146 ticks were collected (comprising 3 species) and tested by real-time PCR (RT-PCR) for the presence of SFGR DNA. Of 127 *A. ovale* ticks, 18 (14.2%) were positive for *R. africae*; 5/12 (41.7%) of *A. sculptum* were positive for *R. amblyommatis*; and 1/7 (14.3%) of *A. triste* ticks were positive for unknown rickettsial DNA [37]. This was the first report of SFGR group *R. africae* in Nicaragua. The second study, also conducted in 2013, tested ticks collected from dogs at various animal shelters in the western portion of the country. The authors tested 316 pools of ticks (1023 individual ticks) through quantitative PCR (qPCR) for the presence of *Rickettsia* spp. DNA, then tested all positive pools for targeted *Rickettsia* spp. In total, 22/316 (7%) of the pools were positive for *Rickettsia* DNA, and 1/199 (0.5%) *R. sanguineus* and 1/3 (33.3%) *A. ovale* ticks were positive for *R. amblyommatis* [38]. The same investigators from the initial 2013 study expanded their investigation to testing the community dogs for evidence of SFGR infection in 2016. The investigators tested 77 dogs in total from three communities using IFA, reporting a prevalence of antibodies to *R. rickettsii* ranging from 70–92.6% [39]. In all three of these studies, the authors made mention of the proximity of these dogs to humans, increasing the risk for ectoparasite and pathogen exposure as risk factors for human disease [37,38,39].

Lastly, a review article conducted in 2019 cites an additional report of SFGR group *A. amblyommatis* in collected *A. ovale* tick from companion animals in Nicaragua; however, no number of ticks collected or percentage of infection was given [40].

## 4. Discussion

Documentation of SFGR *Rickettsia* in El Salvador, Guatemala, Nicaragua, and Honduras dates back to the early 1970s. Of 17 distinct reports, 10 investigated human or animal serology, and 8 investigated the presence of rickettsial bacteria through PCR molecular testing (1 study investigated both serology and tick testing, so is included twice). Common risk factors mentioned in the majority of these studies included contact with animals and spending time outdoors. Comparatively, Costa Rica, Panama, and Belize contribute 54 articles relating to SFGR *Rickettsia*, indicating that there is a distinct dichotomy in the knowledge of these pathogens in humans, animals, and ticks from Central American countries that could be related to socioeconomic factors. Although we had anticipated all three “High” and “Very high” HDI-scored countries (Panama, Costa Rica, and Belize) to have far more articles compared to those scored as “Medium”, only Panama and Costa Rica are noticeably set apart from the rest of the region. Of note is the fact that the size of Belize’s population is much smaller in comparison, and this may contribute to this lack of published articles. We conclude that those countries scored as “Very high” seem to have far greater articles regarding these neglected bacterial pathogens, supporting our hypothesis.

Although there are far fewer articles published from El Salvador, Guatemala, Honduras, Nicaragua, and Belize, we do not believe that this indicates less circulation of SFGR *Rickettsia* in the environment and animal hosts in these countries. Instead, we believe that this lack of published articles indicates a lack of knowledge, awareness, or public health response regarding ticks and tick-borne disease (especially SFGR), potentially increasing the vulnerability of populations in these countries to these vector-borne diseases. This is further supported by the small proportion of authors from the original country of concern for each manuscript for El Salvador, Guatemala, Nicaragua, and Honduras. For example, in a recent conversation with Dr. Amaury Morales Landrove, the Infectious Disease Director for the El Salvador Ministry of Health, it was noted that *Rickettsia* have long been suspected in El Salvador; however, limited funding and political prioritization of other pathogens has rendered SFGR a blind spot in their national surveillance network. This is not a situation unique to El Salvador, as it is well known that these lowest-scoring countries suffer grave poverty, higher numbers of marginalized populations, and higher rates of other NTDs [12,13,41]. In addition, comparing the year of first publication regarding SFGR in each country, Panama and Costa Rica documented their first outbreaks of rickettsial disease in the early 1950s, and the rest of the Central American countries were not publishing anything similar until nearly 20–40 years (Nicaragua, Honduras, and El Salvador) or 60 years later (Belize and Guatemala) (Supplementary Table S1). Although not classically identified as an NTD, SFGR bacteria arguably meet the definition of an NTD given the high rate of potentially severe or fatal complications in disproportionately poor and marginalized communities [10]. As exemplified by our conversation with Dr. Landrove, these lower-scoring HDI countries have constrained public health resources, and thus funding may not be available for tick and tick-borne disease surveillance after focusing efforts on control for mosquito-borne diseases, such as malaria or dengue [24].

As the world continues to change through natural cycles of climate change and human-induced changes from carbon emissions, deforestation, urbanization, and travel, it is anticipated that vector-borne disease ranges will expand globally, including tick-borne disease. In Central America, urbanization and deforestation are happening on a larger scale in than many developed nations [14,42]. Additionally, deforestation occurs disproportionately in the region, where the least-developed countries—Honduras, Nicaragua, Guatemala, Belize, and El Salvador—experience deforestation at a significantly higher rate than Panama and Costa Rica [42]. We anticipate that tick-borne SFGR pathogens will continue to cause morbidity and mortality in this region, with disproportionate effects in those most vulnerable and least-developed countries. As these neglected pathogens are both a cause and consequence of poverty and low human development, we encourage public health officials to try to unearth the burden of SFGR in the Central American region, especially in low-HDI countries. Increased awareness of circulating *Rickettsia* pathogens in the environment will lead to better understanding of epidemiological risk, and ultimately, the development of prevention efforts to stop disease transmission.

## Figures and Tables

**Table 1:** Human development and poverty indicators in Central America.

Country	Human Development Index (HDI) Rank ^1^	HDI Score ^1,†^	Population ^2^	% Child Malnutrition (<5 Years Old) ^1^	% In or Vulnerable to Multidimensional Poverty ^1^	% Below National Poverty Line ^1^	% Living at Less than USD 2/Day (Year of Estimate) ^3^
Panama	57	0.815	4.3 million	19.0	N/A	1.2	1.2 (2019)
Costa Rica	62	0.810	5.2 million	5.6	2.4	1.0	2.1 (2020)
Belize	110	0.716	0.4 million	15.0	9.0	N/A	N/A
El Salvador	124	0.673	6.6 million	13.6	11.6	22.8	1.3 (2019)
Guatemala	127	0.663	17.7 million	46.7	32.3	59.3	8.8 (2014)
Nicaragua	128	0.660	6.3 million	17.3	19.0	24.9	3.4 (2014)
Honduras	132	0.634	9.5 million	22.6	29.1	48.3	14.8 (2019)

^1^ From the United Nations Human Development Programme [21]. ^†^ HDI scores: low (<0.550), medium (0.550–0.699), high (0.700–0.799), very high (≥0.800) [17]. ^2^ From the Central Intelligence Agency [22]. ^3^ From the World Bank [23].

**Table 2:** Comparison of SFGR *Rickettsia*-related articles from all Central American countries.

Country	Number of Articles
Panama	24
Costa Rica	27
Belize	3
El Salvador	5
Guatemala	1
Nicaragua	6
Honduras	5

## Data Availability

The data presented in this study are available on request from the corresponding author.

## Supplementary Materials

The following supporting information can be downloaded at: https://www.mdpi.com/article/10.3390/insects13080674/s1, Table S1: Additional Metrics on SFGR *Rickettsia*-Related Articles from all Central American Countries.
