# Supplementary material for: Spotted Fever Group Rickettsioses in Central America: The Research and Public Health Disparity among Socioeconomic Lines
Source: Insects. 2022 Jul 27;13(8):674. doi: 10.3390/insects13080674 (PMC9332791; doi:10.3390/insects13080674)
Supplement: Supplementary file 1 [file insects-13-00674-s001.zip › insects-1820517-supplementary.pdf]

Supplementary Table S1. Additional Metrics on SFGR *Rickettsia*-Related Articles from all Central American Countries

Country	Country(ies) of Foreign Collaborators	Average Percentage of Authors from Original Country	Year of Publication
Panama	Brazil, Chile, Colombia, Mexico, South Africa, Uruguay, USA	71.7	1950, 1952, 1953, 1956, 1975, 2007, ’09, ’11, ’12, ’13, ’15, ’16, ’17, ’18, ’20, ’21
Costa Rica	Brazil, Czech Republic, Germany, Guatemala, Italy, Panama, Mexico, Spain, USA	74.4	1952, 1971, 1979, 1985, 1986, 2008, ’11, ’12, ’13, ’14, ’15, ’16, ’17, ’18, ’19
Belize	Brazil, Canada, Grenada, USA	16.7	2014, ’16, ‘17
El Salvador	Austria, Brazil, France, Russia, Slovak Republic, Spain, USA	25.3	1993, 1996, 2012, ’14, ‘21
Guatemala	USA	36.4	2013
Nicaragua	Costa Rica, Germany, Panama, USA	12.4	1971, 2016, ’17, ‘18
Honduras	Brazil, Czech Republic, Panama, USA	10.0	1971, 2009, ’10, ’15, ‘21
